# A Novel Set of Immune-associated Gene Signature predicts Biochemical Recurrence in Localized Prostate Cancer Patients after Radical Prostatectomy

**DOI:** 10.7150/jca.51059

**Published:** 2021-05-01

**Authors:** Jiao-chen Luan, Qi-jie Zhang, Kai Zhao, Xiang zhou, Liang-yu Yao, Tong-tong Zhang, Teng-yue Zeng, Jia-dong Xia, Ning-hong Song

**Affiliations:** 1 Department of Urology, The First Affiliated Hospital of Nanjing Medical University, Nanjing, China.; 2 The Affiliated Kezhou People's Hospital of Nanjing Medical University, Kezhou, Xinjiang, China.

**Keywords:** immune-associated gene signature, prostate cancer, biochemical recurrence, radical prostatectomy, prognosis

## Abstract

**Background:** Decision-making regarding biochemical recurrence (BCR) in localized prostate cancer (PCa) patients after radical prostatectomy (RP) mainly relies on clinicopathological parameters with a low predictive accuracy. Currently, accumulating evidence suggests that immune-associated genes (IAGs) play irreplaceable roles in tumorigenesis, progression and metastasis. Considering the critical role of immune in PCa, we therefore attempted to identify the novel IAGs signature and validate its prognostic value that can better forecast the risk for BCR and guide clinical treatment.

**Methods:** RNA-sequencing and corresponding clinicopathological data were downloaded from the Gene Expression Omnibus (GEO) database and the Cancer Genome Atlas (TCGA) database. Weighted gene co-expression network analysis (WGCNA) was utilized to screen out the candidate module closely related to BCR, and univariate and LASSO Cox regression analyses were performed to build the gene signature. Kaplan-Meier (KM) survival analysis, time-dependent receiver operating curve (ROC), independent prognostic analysis and nomogram were also applied to evaluate the prognostic value of the signature. Besides, Gene ontology analysis (GO), Kyoto encyclopedia of genes and genomes (KEGG) and gene set enrichment analysis (GSEA) were used to explore potential biological pathways.

**Results:** A total of six IAGs (SSTR1, NFATC3, NRP1, TUBB3, IL1R1, GDF15) were eventually identified and used to establish a novel IAGs signature. The Kaplan-Meier analysis revealed that patients with low-risk scores had longer recurrence-free survival (RFS) than those with high-risk scores in both GSE70769 and TCGA cohorts. Further, our signature was also proven to be a valuable independent prognostic factor for BCR. We also constructed a nomogram based on the gene signature and related clinicopathologic features, which excellently predict 1-year, 3-year and 5-year prognosis of localized PCa patients after RP. Moreover, functional enrichment analysis demonstrated the vital biological processes, and stratified GSEA revealed that a crucial immune-related pathway (T cell receptor signaling pathway) was notably enriched in the high-risk group.

**Conclusions:** We successfully developed a novel robust IAGs signature that is powerful in BCR prediction in localized PCa patients after RP, and created a prognostic nomogram. In addition, the signature might help clinicians in selecting high-risk subpopulation, predicting survival status of patients and promoting more individualized therapies than traditional clinical factors.

## Introduction

Worldwide, prostate cancer (PCa) ranks the second most commonly diagnosed malignancies in males 1, 2. According to the latest cancer statistics, it is predicted that approximately 192,000 novel PCa patients will be diagnosed and more than 33,000 deaths will appear in the United States in 2020, which account for 21% for incidence and 10% for mortality in all tumors 2. Although radical prostatectomy (RP) is proved to be the primary and effective treatment for clinical localized PCa patients 3-5, approximately 20% of them develop biochemical recurrence (BCR) and eventually progress to castration-resistant prostate cancer (CRPC) 6-8. For these patients, more undergo personalized adjuvant therapy (radiation therapy, chemotherapy, androgen deprivation therapy) or even intensive multimodal therapy 7, 9, 10. Nevertheless, some patients have indolent prostate cancer, which can be followed without immediate treatment and had few effects on living quality. Therefore, to avoid unnecessary overtreatment of indolent disease, it is of great importance to distinguish between these patients for the improvement of prognosis.

As we known, the prognosis of PCa patients is closely related to prostate-specific antigen (PSA), Gleason score (GS) and clinical TNM stage 11-13. Among these clinicopathologic factors, GS is a dominant prognostic parameter. However, due to the sampling error and subjectivity in estimating PSA, GS and TNM stage, patients with similar clinicopathologic features may develop into opposite outcomes. Hence, better prognostic markers are needed to identify patients with high-risk of BCR for the management of localized PCa patients undergoing RP clinically. Nowadays, gene expression signatures turn out to have prognostic value in various forms of cancer, which not only emerge as gene molecular signature to increase the prognostic accuracy, but also help researchers in boosting studies of novel therapy methods 14-16. Recently, some researchers have demonstrated that immune-associated genes (IAGs) play a vital role in the genesis and development of prostate cancer 17. Hussein, et al found that different kinds of immune cells, such as natural killer cells, CD4+ and CD8+T-cells, dendritic cells and tumor-associated macrophages were detected in prostate cancer tissues 18. For instance, T-cell infiltration has been illustrated to be related to tumor progression and cancer-specific survival (CSS) in both localized and metastatic patients 19, 20. Natural killer (NK) cells seem to be connected with a lower risk of progression 21. Tumor-associated macrophages appear to be associated with aggressive pathologic features and recurrence after prostatectomy 22. Concerning immunotherapy, a variety of antigen delivery systems serve as feasible and promising immunotherapeutic agents against prostate cancer 23-25. Sipuleucel-T, the first approved by the US FDA for the treatment of men with asymptomatic or minimally symptomatic CRPC, was a landmark in cancer immunotherapy, and such immune resistance likely exists because of immunogenicity of cancer cells and an immunosuppressive tumor microenvironment 23, 26.

However, there are few studies associating IAGs with BCR in localized PCa patients after RP. Considering the crucial role of the immune system in the prognosis of PCa, we determined to establish novel predictive IAGs signature to improve the risk stratification of biochemical recurrence in PCa patients via employing the high-throughput sequencing results and downloading clinical data from the Gene Expression Omnibus (GEO, https://www.ncbi.nlm.gov/geo/) database and the Cancer Genome Atlas (TCGA, https://portal.gdc.cancer.gov/). The results might offer a more powerful tool for clinicians to make individualized and precise medical decisions and have a better understanding of possible molecular mechanisms of BCR.

## Materials and Methods

### Acquisition of the eligible sample datasets and IAGs

In our study, 471 localized PCa patients following RP and full-scale clinical parameters from two independent cohorts were included. One was from the Gene Expression Omnibus (GEO), and the other was from the Cancer Genome Atlas (TCGA) cohort. The RNA-seq data of GSE70769 were produced with Illumina HumanHT-12 V4.0 Array, for 92 samples with full-scale clinical information. Besides, RNA-sequencing (RNA-seq) expression profile of 379 patients was downloaded from the TCGA dataset (**Table S1**). Inclusion and exclusion criteria of eligible patients: The inclusion criteria were as follows: (I) biospecimens were concentrated from localized prostate cancer patients after radical prostatectomy; (II) clinicopathological characteristics, such as Gleason score (GS), clinical stage T (cT), prostate-specific antigen (PSA) or surgical margins (SM) were contained; (III) the outcomes (biochemical recurrence (BCR) or BCR-free (BCR-F)) of samples were included; The exclusion criteria were as follows: (I) patients also following chemotherapy or radiotherapy in addition to the radical prostatectomy. In our study, GSE70769 was utilized as a training set, while the TCGA database was applied to validate. The whole RNA-seq data and microarray were log2 transformed and normalized with the manufacture-provided R packages. A collection of immune-associated genes (IAGs) were downloaded from the Immunology Database and Analysis Portal (ImmPort, https://immport.niaid.nih.gov) 27.

### Weighted Gene Co-Expression Network Analysis

In accordance with the microarray data from the training database, the weighted gene co-expression network analysis (WGCNA) was performed to establish a scale-free co-expression network 28. First, based on the expression of IAGs, a hierarchical clustering analysis of PCa patients with different clinicopathological features was used to remove outlier patients. Next, according to the Pearson's correlation coefficient between IAGs, the suitable soft threshold power (β) was selected to construct the scale-free network, and β = 3 (score free R^2 = 0.89) was considered to ensure the establishment of the network (**Figure S1B**) in our study. Then, utilizing the topological overlap matrix (TOM)-based dissimilarity measure, with deep-Split of 2 and min-Module size (gene group) of 30 for the IAGs cluster dendrogram, average linkage hierarchical clustering was created. IAGs with similar expression modes were stratified into the same modules by a one-step network construction and module detection. Besides, two parameters were defined. One was gene significance (GS), which was used to quantify the connection between individual IAG and biochemical recurrence-free survival (RFS). The other was module eigengenes (MEs), which served as the first principal component-related module whose value could be regarded to represent the whole IAGs in the module. Under the two parameters, the excellent candidate module that had the highest absolute correlations with RFS was singled out for further analysis.

### Establishment and validation of the risk score model

IAGs deriving from the candidate module was submitted for the univariate Cox regression analysis, and those were significantly associated with biochemical recurrence-free survival (RFS) (p < 0.05) were screened for prognostic signature development. Subsequently, we performed the least absolute shrinkage and selection operator (LASSO) Cox regression analysis to single out the optimal prognostic IAGs 29. Eventually, with individual normalized gene expression value weighted by its LASSO Cox regression coefficients, the risk score algorithm of each PCa patient was established as (exprIAG1 × coefficientIAG1) + (exprIAG2 × coefficientIAG2) + ⋯ + (exprIAGi × coefficientIAGi). Specifically, where i is the number of IAG, coefficientIAGi is the regression coefficient of IAG i, and exprIAGi is the expression value of each candidate IAG i.

Moreover, in light of the median risk score in the training set as the threshold, eligible PCa patients were divided into low- and high-risk groups. The Kaplan‐Meier survival curves were plotted to compare the prognostic difference in RFS between the two subgroups mentioned above. The time-dependent receiver operating characteristic curves (ROC) was generated by using the “SurvivalROC” package, and area under the curve (AUC) values were calculated to estimate the specificity and sensitivity of the risk score. To further determine the predictive ability of our model, the TCGA database was adopted as a validation cohort. The univariate and multivariate Cox proportional hazards regression analyses were performed to assess the BCR outcome predictive performance of our model and other related clinicopathological features.

### Construction of the prognostic nomogram

To offer a clinically quantitative tool to monitor and predict BCR outcomes in PCa patients, a novel nomogram model was established 30, which integrated the IAGs signature and related clinicopathological parameters. The ROC curve was plotted, and the area under the ROC curve (AUC) was subsequently calculated to assess the veracity of the compound nomogram. Besides, to compare the predicted and actual observed BCR results of the compound nomogram, the calibration curves were applied, among which the 45° line represented the top prediction.

### Functional enrichment analysis of IAGs

Gene ontology analysis (GO) functioned as a standard method for noting genes and identifying biological attributes for high-throughput genome data, containing biological process (BP), cellular component (CC) and molecular function (MF). As a knowledge base for systematically analyzing gene functions, Kyoto encyclopedia of genes and genomes (KEGG, http://www.genome.jp/) connected genomic information with higher-order functional details. GO enrichment analysis and KEGG pathway analysis were carried out on the Database for Annotation, Visualization and Integrated Discovery (DAVID) online tool (version 6.8; https://david.ncifcrf.gov/). Both FDR and P-value < 0.05 were regarded as statistically significant.

Then the downloaded gene set enrichment analysis (GSEA, http://www.broadinstitute.org/gsea/index.jsp) was applied to identify the pathways, which were mainly enriched between high-risk and low-risk groups 31. The number of permutations was set to 1000 for each analysis, and the normalized enrichment score (NES) value was calculated for each gene set. The gene size smaller than 15 or larger than 500 was excluded, and a gene set was regarded as the enriched group when nominal NES > 1.5 and p-value < 0.05.

### Statistical analyses

In our study, the Fisher's exact test or Chi‐squared test was executed for categorical variables, and one-way analysis of variance (ANOVA) or Student's t-test for continuous variables. The univariate Cox proportional hazards regression model and LASSO regression analysis were used to calculate the hazard ratio and the regression coefficient. Survival curves were analyzed by the Kaplan‐Meier method utilizing the log-rank test. The Z-score method was applied to normalize risk scores in the training and validation cohort. The SPSS Statistics 26.0 and R software 3.6.3 were performed for all statistical analyses. It was considered significantly different that two-sided P values were less than 0.05.

## Results

### Establishment of a prognostic IAGs signature

**Figure 1:**
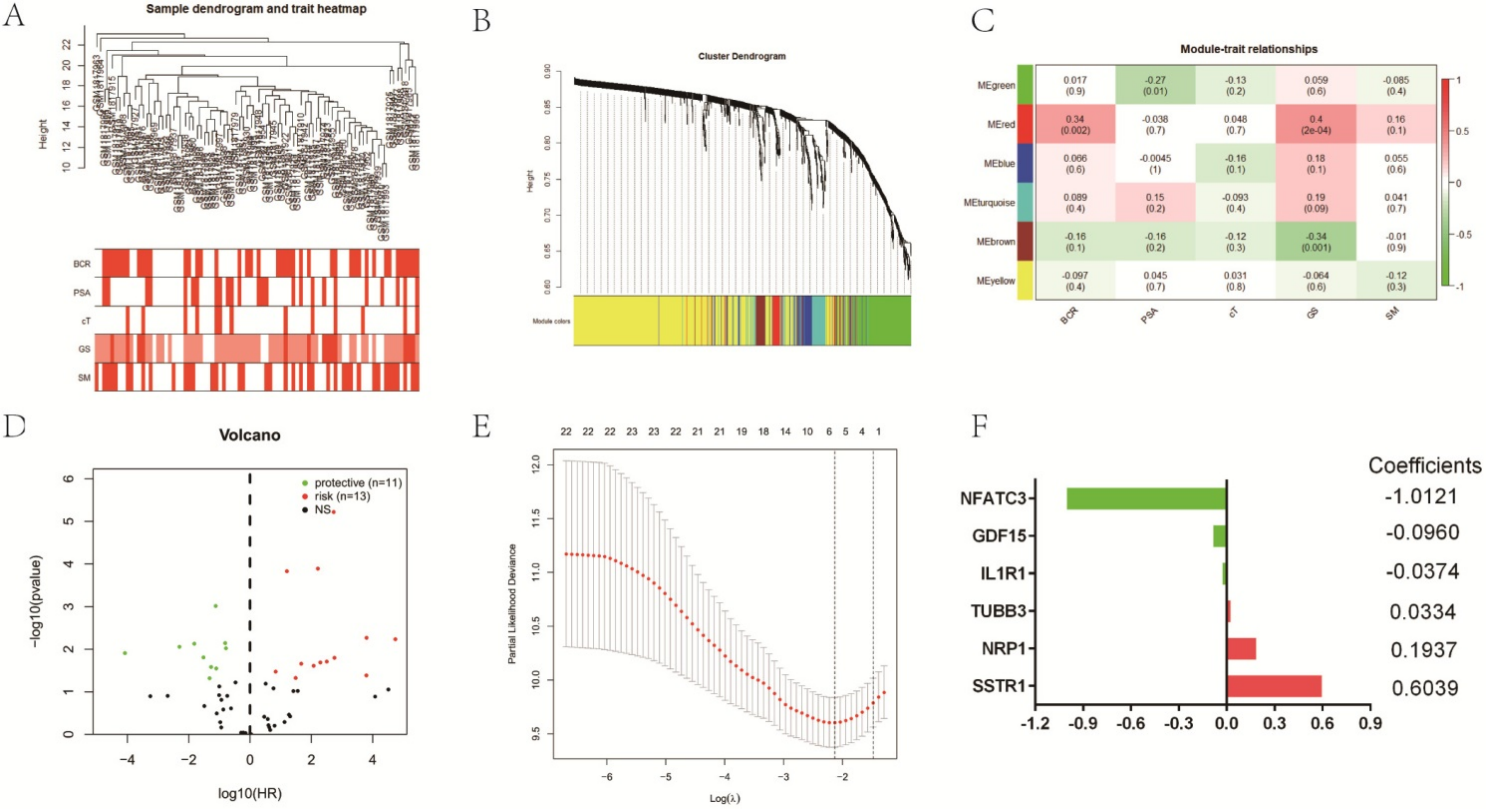
Selection of eligible biomarkers to construct a prognostic immune-associated genes (IAGs) signature. (A) Cluster tree of localized PCa samples in GSE70769, and the color band underneath the tree displaying the numeric values of clinicopathologic features; (B) Cluster Dendrogram showing different IAGs modules; (C) Heatmap visualizing the correlation between the modules and clinicopathologic features; (D) Volcano plot revealing the result of univariate Cox regression analysis; (E) LASSO regression with tenfold cross-validation obtained six prognostic IAGs utilizing minimum lambda value. (F) Distribution of LASSO coefficients of IAGs signature. PCa: prostate cancer; IAGs: immune-associated genes; BCR: biochemical recurrence; LASSO: least absolute shrinkage and selection operator.

To identify the relationship between IAGs and localized PCa patients with different clinicopathologic features, the expression data profile of these 1811 IAGs was transformed into a gene co-expression network utilizing a WGCNA package in the training cohort (**Figure 1**). Sample clustering illustrated that the sample GSM1817916 was an outlier that were excluded from our study (**Figure S1A**), and the other eligible samples along with different BCR, PSA, cT, GS, and SM rates were selected for IAGs expression clustering (**Figure 1A**). With a power of β = 3 set as the optimal soft threshold, a total of six co-expressed modules were identified through a scale-free network establishment method (**Figure 1B and Figure S1B**). Among these co-expressed modules, one module (red) with the highest absolute correlation value with BCR was screened out (p = 0.002 and correlation coefficient = 0.34) (**Figure 1C** and **Figure S1C**). Next, the 58 IAGs from the red module was submitted for the univariate Cox regression analysis. Taking the cut-off value of P < 0.05, 24 IAGs were identified to be significantly linked to the RFS of localized PCa patients (**Figure 1D**). Then, in order to generate the hub candidate IAGs, the LASSO Cox regression analysis was applied to get the optimal lambda value, which came from the minimum partial likelihood deviance (**Figure 1E**). In the end, six critical prognostic IAGs comprising of SSTR1, NFATC3, NRP1, TUBB3, IL1R1 and GDF15 were selected, and their individual nonzero LASSO coefficients were shown in **Figure 1F**. Furthermore, we established a six prognostic IAGs signature by employing the risk score model. The risk score of each sample was calculated according to the following formula:

Risk score = (-1.0121*expression level of NFATC3) + (-0.0960*expression level of GDF15) + (-0.0374*expression level of IL1R1) + (0.0334*expression level of TUBB3) + (0.1937*expression level of NRP1) + (0.6039*expression level of SSTR1)

### IAGs signature is an independent risk factor in localized PCa patients

**Figure 2:**
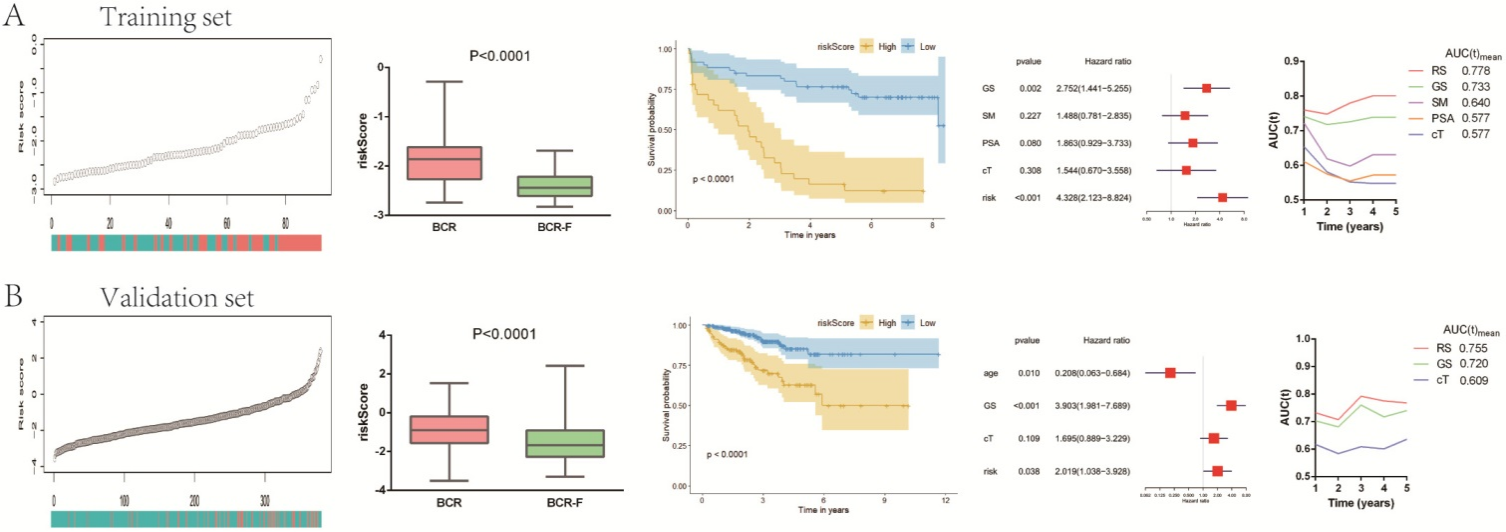
Predictive value of gene signature for BCR in (A) GSE70769 dataset (training cohort) and (B) TCGA database (validation cohort). Risk scores in BCR patients were significantly higher than those with BCR-free. The Kaplan-Meier analysis demonstrated that patients with low risk scores had a significantly longer recurrence-free survival (RFS) than those with low risk scores. The multivariate Cox regression model illustrated that the risk score was an independent prognostic risk factor. The time-dependent receiver operating characteristic (ROC) model showed that the risk score was a powerful predictor for BCR. BCR: biochemical recurrence.

In accordance with the coefficient value of the six IAGs, the risk scores of all samples were calculated and ranked in the training set and the validation set. The risk scores of BCR-free (BCR-F) patients were obviously decreased compared with those of BCR ones in both cohorts. Based on the risk score, patients in the training set were divided into the high-risk and low-risk groups. Kaplan-Meier survival curves displayed that localized PCa patients in the high-risk group had a significantly poorer recurrence-free survival (RFS) compared to those in the low-risk one (p < 0.0001) in the training database (**Figure 2A**). Utilizing the same regression coefficient (β) and algorithm, those findings were subsequently tested in the validation set, which presented the same result as expected, with a significantly longer RFS in the low-risk group (p < 0.0001) (**Figure 2B**). The Cox's regression model was performed to determine whether the signature was an independent factor compared to other clinicopathological factors, such as GS, SM, PSA and age. After the multivariate Cox regression analysis, the results demonstrated that the IAGs signature was an independent prognostic parameter, which was significantly connected with RFS (training set: HR = 4.328, 95%CI 2.123-8.824, P < 0.001; validation set: HR = 2.019, 95%CI 1.038-3.928, P = 0.038) (**Figure 2A** and** 2B**). To estimate the prognostic power of the IAGs signature, the time-dependent ROC analyses were used, and the area under curve (AUC) values were calculated. We found that the average AUC value of the risk score for RFS was 0.778 at five years follow-up in the training set, which was significantly higher than those of GS (AUC = 0.733), SM (AUC = 0.640), PSA (AUC = 0.577) and cT (AUC = 0.577). Similarly, in the validation set, average AUC value of the risk score (AUC = 0.755) was also considerably higher than those associated with GS (AUC = 0.720) and cT (AUC = 0.609) (**Figure 2A** and **2B**). The outcomes mentioned above suggested that the IAGs signature served as a more powerful predictor for BCR than other clinical features in localized PCa patients.

In addition, we normalized the risk score to Z-score for both TCGA and GSM1817916 cohorts. The results indicated that compared to BCR-free (BCR-F) patients, Z-score was significantly higher in BCR patients. What's more, with the extension of BCR time, Z-score also has a tendency to increase gradually (**Figure S2**).

### Construction and validation of the prognostic nomogram based on the IAGs signature

**Figure 3:**
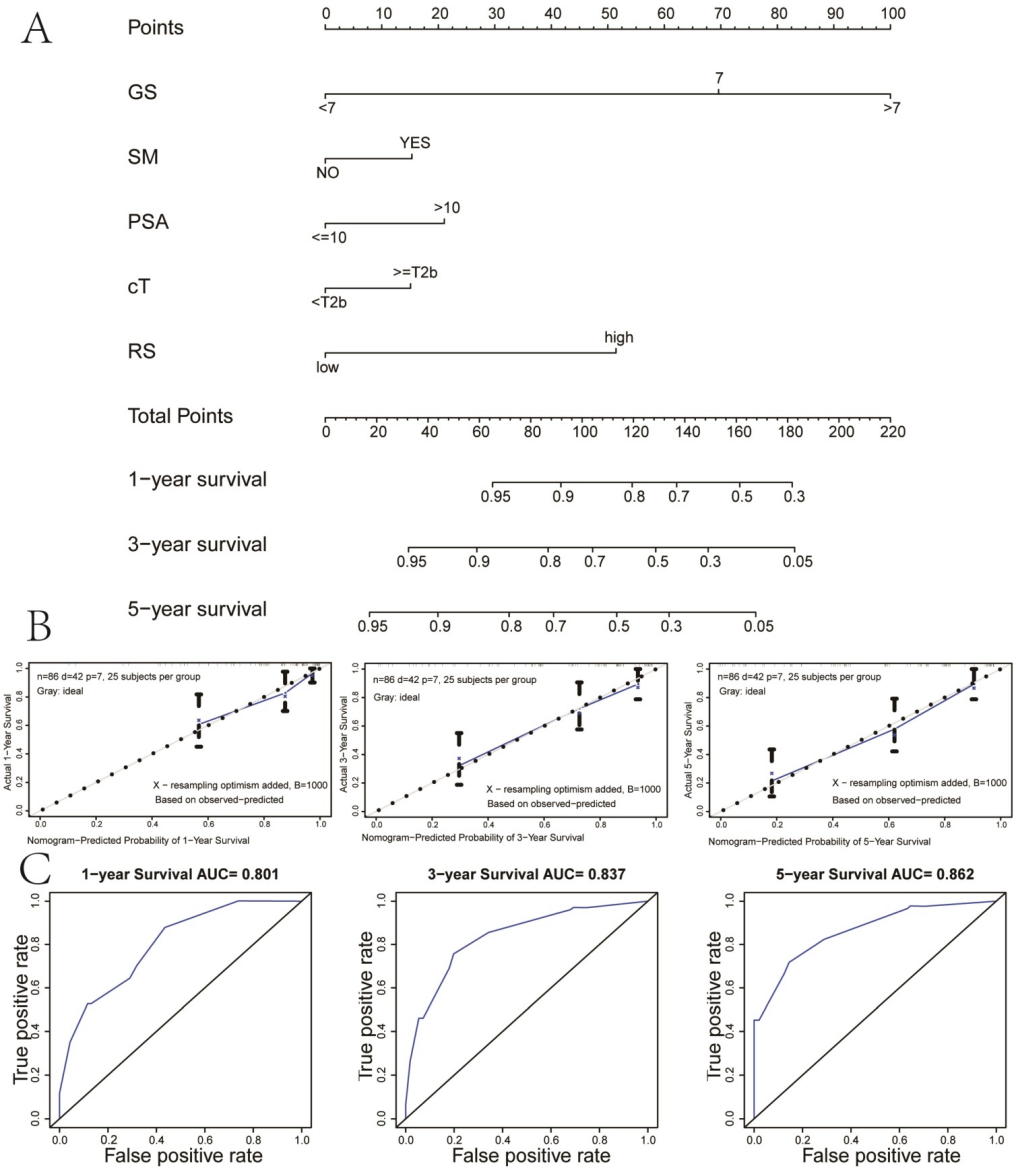
Nomogram and calibration plots based on our established signature and clinical variables. (A) Nomogram for predicting probabilities of localized PCa patients with 1-, 3‐, 5‐year survival; (B) The calibration plot of the nomogram for agreement test between 1-, 3- and 5-year survival prediction and actual result; (C) The receiver operating characteristic (ROC) curves of the nomogram to predict 1-, 3- and 5-year survival. PCa: prostate cancer.

To provide a clinically quantitative method to monitor and predict the prognosis of localized PCa patients undergoing BCR after RP, we constructed a novel prognostic nomogram that integrated the risk score, GS, SM, cT and PSA. A total point could be calculated by adding the scores of five variables in the nomogram, among which survival probabilities could be estimated easily. The results showed that the prognostic nomogram could better predict 1-, 3- and 5-year RFS of localized PCa patients (**Figure 3A**). In the training set, the AUC value of 1-year, 3-year and 5-year RFS of the new nomogram were 0.801, 0.837 and 0.862, respectively, which presented the excellent prognostic power in predicting RFS (**Figure 3B**). The calibration plots of our nomogram for survival prediction suggested an excellent conformity between the expected and the observed outcomes (**Figure 3C**).

### Identification of biological pathways of IAGs in the candidate module related to BCR

**Figure 5:**
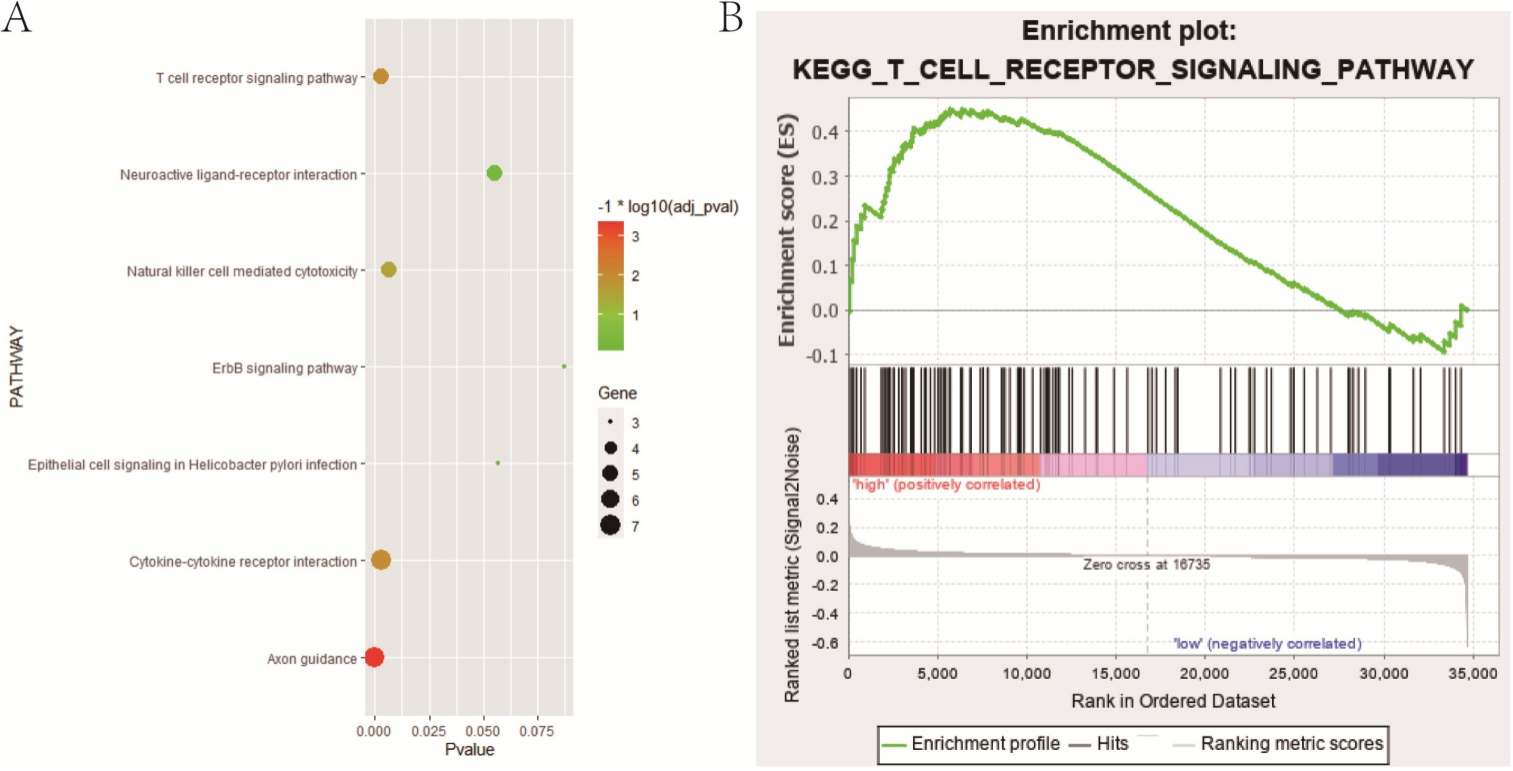
Functional enrichment analysis. (A) Bubble chart indicating KEGG pathways that 58 IAGs in candidate module (red module) were mainly enriched in; (B) One important immune-related pathway, namely T cell receptor signaling pathway enriched in the high-risk group based on stratified GSEA. IAGs: immune-associated genes; KEGG, Kyoto encyclopedia of genes and genomes; GSEA, gene set enrichment analysis.

The GO enrichment and KEGG pathway analysis were performed to explore the potential functional and molecular mechanisms of IAGs in the candidate module (red module). GO enrichment analysis demonstrated that the candidate IAGs were mainly enriched in the biological processes (BP) connected with regulation of cell proliferation, response to wounding, cell surface receptor linked signal transduction, secretion by cell and antigen processing and presentation, etc. Regarding the cellular component (CC) analysis, we found that the candidate IAGs were notably associated with acellular region, extracellular space, integral to plasma membrane, intrinsic to plasma membrane and extracellular region part, etc. In terms of the molecular function (MF), the candidate IAGs were significantly involved in cytokine activity, cytokine binding, peptide binding, peptide receptor activity, peptide receptor activity and G-protein coupled, etc. Moreover, KEGG pathway analysis revealed that the candidate IAGs were mainly enriched in axon guidance, T cell receptor signaling pathway, cytokine-cytokine receptor interaction, natural killer cell mediated cytotoxicity and neuroactive ligand-receptor interaction, etc. (**Figure 5A** and **Table 1**).

Additionally, we carried out the gene set enrichment analyses (GSEA) to identify the potential biological processes between high- and low-risk groups in the training cohort (**Table 2**). Intriguingly, stratified GSEA demonstrated that an important immune-related pathway, namely T cell receptor signaling pathway, was notably enriched in the high-risk group (**Figure 5B**).

### Verification of the expression and prognostic value of IAGs in the signature

**Figure 4:**
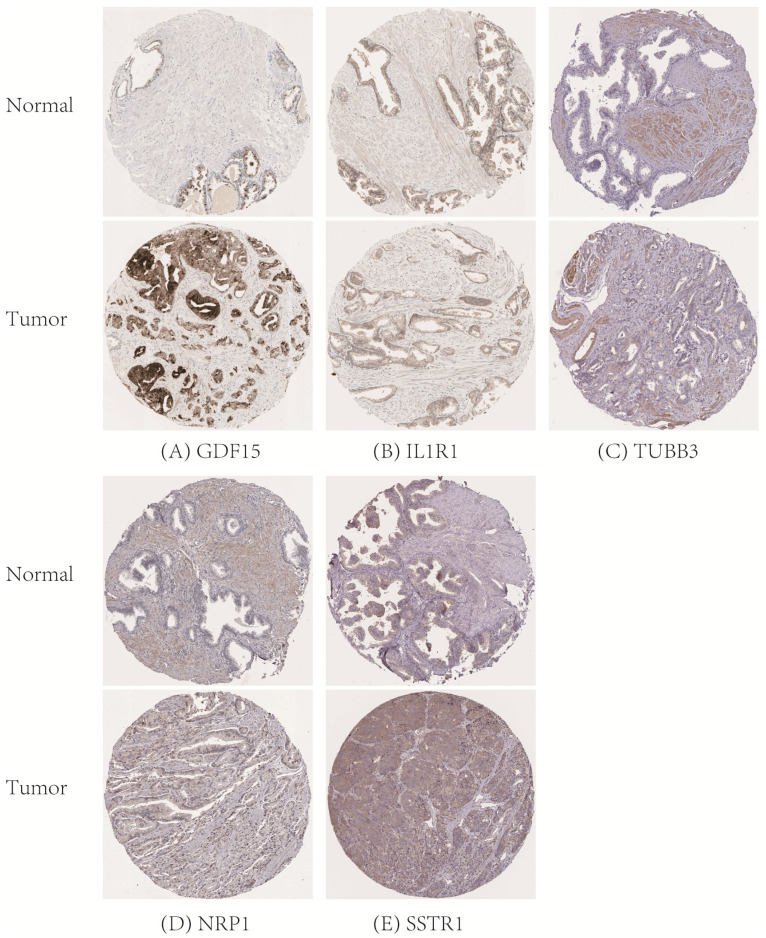
Verification of hub IAGs expression in prostate cancer and normal prostate tissue utilizing the Human Protein Atlas (HPA) database. (A): GDF15; (B): IL1R1; (C): TUBB3; (D): NRP1; (E): SSTR1. IAGs: immune-associated genes.

Then the expression profiles of the six hub IAGs between tumor and normal tissue were presented in **Figure S3**, which displayed that GDF15, TUBB3 and SSTR1 were significantly upregulated in prostate cancer, while NFATC3 and IL1R1 were significantly downregulated when compared with normal tissue (p < 0.05). However, the expression level of NRP1 was not significantly different between normal and prostate tissue (p = 0.5305). Moreover, to validate the protein expression of hub IAGs in prostate cancer, immunohistochemistry results from the Human Protein Atlas (HPA) database (http://www.proteinatlas.org/) were utilized, which showed the similar results as expected (**Figure 4**).

## Discussion

Prostate cancer is a common malignancy among elderly males in the world 2. Unfortunately, approximately 27 to 53% of PCa patients develop local recurrence or distant metastasis within ten years after surgery 32-34. Biochemical recurrence (BCR), defined as a re-increase in prostate-specific antigen (PSA) above 0.2 µg/L and confirmed by two consecutive elevated values, is a decisive risk factor for distant metastasis, prostate cancer-specific and overall mortality 35, 36. Evidence demonstrated that without a secondary therapy following BCR, about 30% of patients underwent clinically manifested distant metastasis, and 19-27% of patients may suffer prostate-cancer specific mortality within 10 years 37, 38. Therefore, it is highly desirable to stratify localized PCa patients following RP with high-risk of BCR, which may provide more frequent monitoring, early intervention and even decision-making for adjuvant therapy. Instead of utilizing traditional tumor risk stratification tools based on clinical parameters, our study aimed at finding novel biomarkers for a more precise prediction of BCR.

During the era of cancer immunotherapy, PCa is the first malignancy to demonstrate improved survival with a cancer-specific vaccine, proving that it is an immune responsive disease 39, 40. The role of immune surveillance in PCa has been extensively explored, and some studies have illustrated that compared with the control group, the overall survival rate of patients got improved after treatment with relevant immunologically active substances 17, 40, 41. Therefore, the use of immune-associated genes (IAGs) as a prognostic factor for PCa is convincing. Our study is the first research to establish a prognostic model based on IAGs in patients with localized prostate cancer.

In our study, we singled out six immune-associated genes (SSTR1, NFATC3, NRP1, TUBB3, IL1R1, GDF15) to construct the BCR predictive model based on the GSE70769 dataset (training cohort) and validate it in the TCGA database (validation cohort). We performed WGCNA to identify the candidate gene module (red module) that was most significantly connected with BCR. Then, the univariate Cox regression analysis and LASSO regression analysis were utilized to select six hub genes from the red module. On the basis of six IAGs, we established the predictive signature and calculated the risk score of each patient according to the formula. Further, the enrolled patients were divided into high- and low-risk groups based on the median risk score value, and the Kaplan-Meier survival analysis demonstrated that patients with low risk scores had a significantly longer recurrence-free survival (RFS) than those with high risk scores in both TCGA and GSE70769 cohorts. Univariate and multivariate Cox regression analysis displayed that the risk score was an independent prognostic factor for BCR. What's more, we performed the time-dependent ROC analysis, which illustrated that our model exhibited the excellent predictive performance in both the training and validation datasets. The efforts above underscored that our six IAGs signature had the satisfactory sensitivity and specificity in predicting BCR of localized PCa patients after RP.

In addition, to provide a quantitative tool for clinicians to predict BCR and improve risk stratification, a compound nomogram was established that integrated the risk score and four relative clinicopathological parameters (GS, SM, PSA, and cT). In the calibration plots of our novel prognostic nomogram, greatly satisfactory conformity was found between the predicted and observed outcomes. The results of time-dependent ROC analysis showed that the novel nomogram had the wonderful prognostic power in predicting RFS, with an AUC of 0.862 after 5-year follow-up. Therefore, our prognostic nomogram might better help clinicians monitor and predict the 1-, 3- and 5-year RFS of localized PCa patients following RP, and provide better individualized therapy than original clinical protocols.

Some IAGs involved in our signature have been investigated to play critical roles in tumors, even in prostate cancer. For example, GDF15, namely a divergent TGF-beta superfamily cytokine, slowed the growth of PCa by irritating tumor immunity. And the overexpression of GDF15 was related to an increased number of CD8 T cells, an increased number of CD8+CD11c+ T cells and a reduced proportion of “exhausted” CD8+PD1+ T cells 42, 43. NFATC3 was widely associated with the progression and prognosis of multiple human cancers, such as colorectal cancer (CRC) 44, gastrointestinal cancer 45, human glioblastoma (hGB) 46, oral/oropharyngeal squamous cell carcinoma (OSCC) 47 and breast cancer 48. Regarding TUBB3, it was reported that TUBB3 overexpressed in castration-resistant prostate cancer (CRPC), and might function as a negative predictor of docetaxel-resistance in metastatic CRPC patients 49. NRP1, known as a regulator of neuronal guidance and angiogenesis, expressed in various malignancies and promoted tumor angiogenesis 50, 51. Yeh, et al. demonstrated that NRP1, serving as a novel androgen-suppressed gene, upregulated during the adaptive response of PCa to androgen-targeted therapies (ATTs), and could be a prognostic biomarker of clinical metastasis and lethal PCa 51. As for IL1R1, Gerashchenko, et al revealed that IL1R1 was downregulated in prostate cancer, and relative expression of IL1R1 was found in tumors with different Gleason score (GS) 52. SSTR1 was linked to androgen receptor (AR) expression and tumor metastasis. Besides, by decreasing cell-proliferation and PSA secretion, SSTR1 exerted a significant pathophysiological role in prostate cancer, and could be served as a novel tool to explore therapeutic targets 53.

As the six IAGs signature demonstrated excellent ability in predicting BCR in PCa patients, the signaling pathways and biological processes also needed to explore in our study. The GO enrichment analysis and KEGG pathway analysis illustrated that IAGs in the candidate module were significantly enriched in T cell receptor signaling pathway, natural killer cell mediated cytotoxicity, regulation of cell proliferation, cell surface receptor linked signal transduction, extracellular region, extracellular space, cytokine activity, cytokine binding, peptide binding and peptide receptor activity, et al. Stratified GSEA indicated that in the high-risk group, biological processes were significantly enriched in T cell receptor signaling pathway, progesterone mediated oocyte maturation, blood vessel endothelial cell proliferation involved in sprouting angiogenesis, cell aggregation, mediator complex, anchored component of plasma membrane, protein deacetylase activity, glutamate receptor activity and so on. It was worth noting that one immune-related pathway, namely T cell receptor signaling pathway was enriched in both analyses. Therefore, we supposed that T cell receptor signaling pathway might make considerable contributions to tumorigenesis, progression and poor prognosis in localized PCa patients after RP, which further showed the high clinical value of our gene signature.

Overall, our IAGs based signature was successfully established and carefully evaluated in the discovery and validation databases. The signature is of vital importance for clinical application, helping predict and monitor the progression and metastasis of prostate cancer. In spite of the merits, several limitations remained to be acknowledged. First, our study was the retrospective design, thus the reliability of our six IAGs signature is needed to verify in multi-center and prospective researches. Second, more and more experiments should be carried out to further investigate the potential biological progress and molecular mechanisms of these IAGs in prostate cancer progression.

## Conclusion

In conclusion, our study identified and established a novel robust IAGs (SSTR1, NFATC3, NRP1, TUBB3, IL1R1, GDF15) signature in predicting BCR for localized PCa patients following RP, which could help clinicians predict patients' recurrence-free survival (RFS) and improve the specific individualized management than original clinical parameters. Moreover, the signature could also serve as an independent prognostic factor, and nomogram based on it showed an excellent predictive efficacy of patients' survival status. However, further studies are expected to validate our prognostic model, and functional researches are required to better understand the molecular mechanisms in prostate cancer.

## Supplementary Material

Supplementary figures and tables.

## Figures and Tables

**Table 1:** GO and KEGG about immune-associated genes (IAGs) in the candidate module (red module) related to biochemical recurrence (BCR)

Items	-logP
***GO***	
**BP**	
regulation of cell proliferation	4.67
response to wounding	3.18
cell surface receptor linked signal transduction	3.14
secretion by cell	3.01
antigen processing and presentation	2.45
**CC**	
extracellular region	5.91
extracellular space	4.36
integral to plasma membrane	4.00
intrinsic to plasma membrane	3.89
extracellular region part	3.69
**MF**	
cytokine activity	4.06
cytokine binding	3.13
peptide binding	2.15
peptide receptor activity	2.04
peptide receptor activity, G-protein coupled	2.04
**KEGG**	
axon guidance	4.16
T cell receptor signaling pathway	2.52
cytokine-cytokine receptor interaction	2.51
natural killer cell mediated cytotoxicity	2.19
neuroactive ligand-receptor interaction	1.26

GO: Gene ontology; KEGG: Kyoto encyclopedia of genes and genomes; BP: biological process; CC: cellular component; MF: molecular function.

**Table 2:** Gene set enrichment analyses (GSEA) between high- and low-risk groups in the training cohort (terms enriched in high-risk group)

Items	Size	NES	P-value
**C2 KEGG**			
KEGG_DORSO_VENTRAL_AXIS_FORMATION	24	1.70	0.004
KEGG_AMYOTROPHIC_LATERAL_SCLEROSIS_ALS	52	1.68	0.007
KEGG_UBIQUITIN_MEDIATED_PROTEOLYSIS	129	1.67	0.009
KEGG_PROGESTERONE_MEDIATED_OOCYTE_MATURATION	84	1.66	0.010
KEGG_T_CELL_RECEPTOR_SIGNALING_PATHWAY	106	1.57	0.033
**C5 BP**			
GO_BLOOD_VESSEL_ENDOTHELIAL_CELL_PROLIFERATION_INVOLVED_IN_SPROUTING_ANGIOGENESIS	49	1.80	0.002
GO_POSITIVE_REGULATION_OF_CELL_MIGRATION_INVOLVED_IN_SPROUTING_ANGIOGENESIS	36	1.80	0.002
GO_CELL_AGGREGATION	20	1.75	0.004
GO_NEGATIVE_REGULATION_OF_ENDOTHELIAL_CELL_APOPTOTIC_PROCESS	33	1.66	0.006
GO_POSITIVE_REGULATION_OF_BLOOD_VESSEL_ENDOTHELIAL_CELL_MIGRATION	74	1.62	0.004
**C5 CC**			
GO_CULLIN_RING_UBIQUITIN_LIGASE_COMPLEX	135	1.69	0.002
GO_MEDIATOR_COMPLEX	35	1.69	0.006
GO_ANCHORED_COMPONENT_OF_PLASMA_MEMBRANE	54	1.65	0.002
GO_CATENIN_COMPLEX	28	1.64	0.008
GO_U2_SNRNP	21	1.63	0.028
**C5 MF**			
GO_PROTEIN_DEACETYLASE_ACTIVITY	29	1.93	0.002
GO_NAD_DEPENDENT_PROTEIN_DEACETYLASE_ACTIVITY	15	1.88	0.002
GO_GLUTAMATE_RECEPTOR_ACTIVITY	27	1.85	0.007
GO_VOLTAGE_GATED_CALCIUM_CHANNEL_ACTIVITY	46	1.74	0.008
GO_UBIQUITIN_LIKE_PROTEIN_CONJUGATING_ENZYME_ACTIVITY	38	1.71	0.002

GO: Gene ontology; KEGG: Kyoto encyclopedia of genes and genomes; BP: biological process; CC: cellular component; MF: molecular function.

## Abbreviations

BCR: biochemical recurrence
RP: radical prostatectomy
IAGs: immune-associated genes
PCa: prostate cancer
GEO: Gene Expression Omnibus
TCGA: the Cancer Genome Atlas
WGCNA: Weighted gene co-expression network analysis
KM: Kaplan-Meier
ROC: receiver operating curve
GO: Gene ontology analysis
KEGG: Kyoto encyclopedia of genes and genomes
GSEA: gene set enrichment analysis
RFS: recurrence-free survival
CRPC: castration-resistant prostate cancer
cT: clinical T
SM: surgical margins
BCR-F: biochemical recurrence-free
LASSO: least absolute shrinkage and selection operator
AUC: area under the curve
BP: biological process
CC: cellular component
MF: molecular function
NES: normalized enrichment score
HPA: Human Protein Atlas
