# Supplementary material for: A Novel Set of Immune-associated Gene Signature predicts Biochemical Recurrence in Localized Prostate Cancer Patients after Radical Prostatectomy
Source: J Cancer. 2021 May 1;12(12):3715–25. doi: 10.7150/jca.51059 (PMC8120173; doi:10.7150/jca.51059)
Supplement: Supplementary file 1 — Supplementary figures and tables. [file jcav12p3715s1.pdf]

Figure S1. WGCNA analysis in PCa. (A) Sample clustering was applied to exclude outliers; (B) Analysis of the scale-free fit index for various soft-thresholding powers (left) and the mean connectivity for various soft-thresholding powers (right); (C) Heatmap visualizing the correlation between the modules and biochemical recurrence (BCR). PCa: prostate cancer.

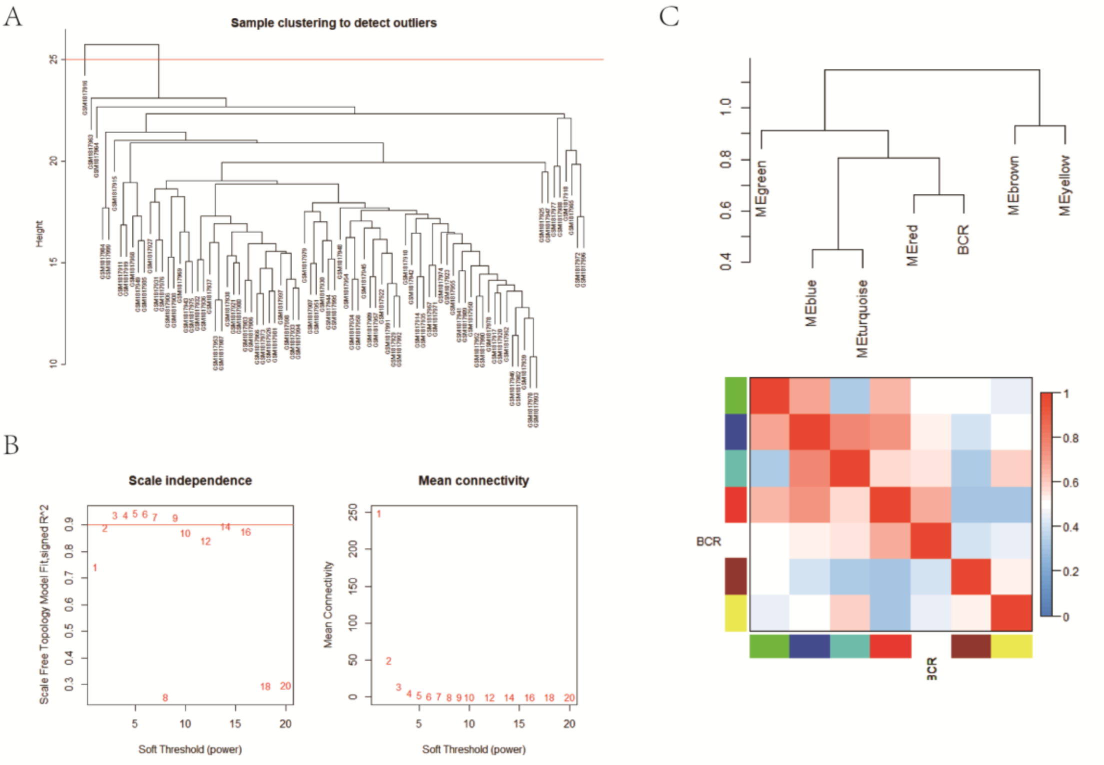

Figure S2. Z-score in BCR patients was significantly higher compared with BCR-free (BCR-F) patients, and had a tendency to increase with the extension of BCR time. BCR: biochemical recurrence.

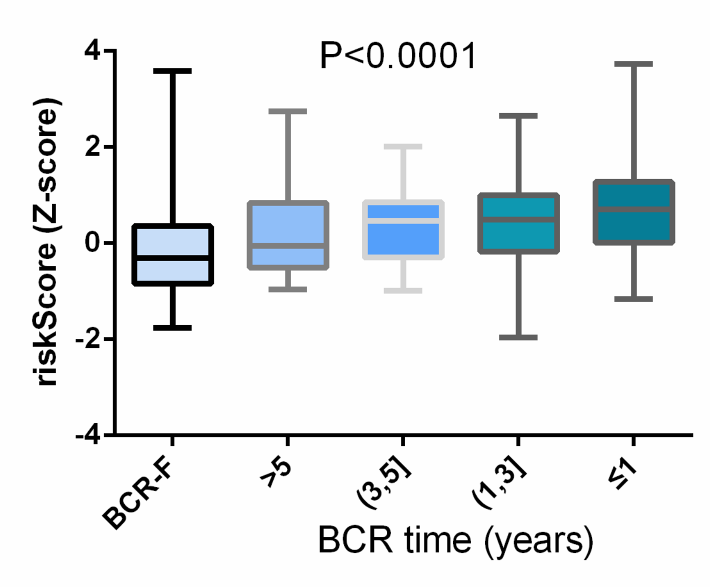

Figure S3. The expression level of IAGs (SSTR1, NFATC3, NRP1, TUBB3, IL1R1, GDF15) in the signature in prostate cancer and normal prostate tissue.

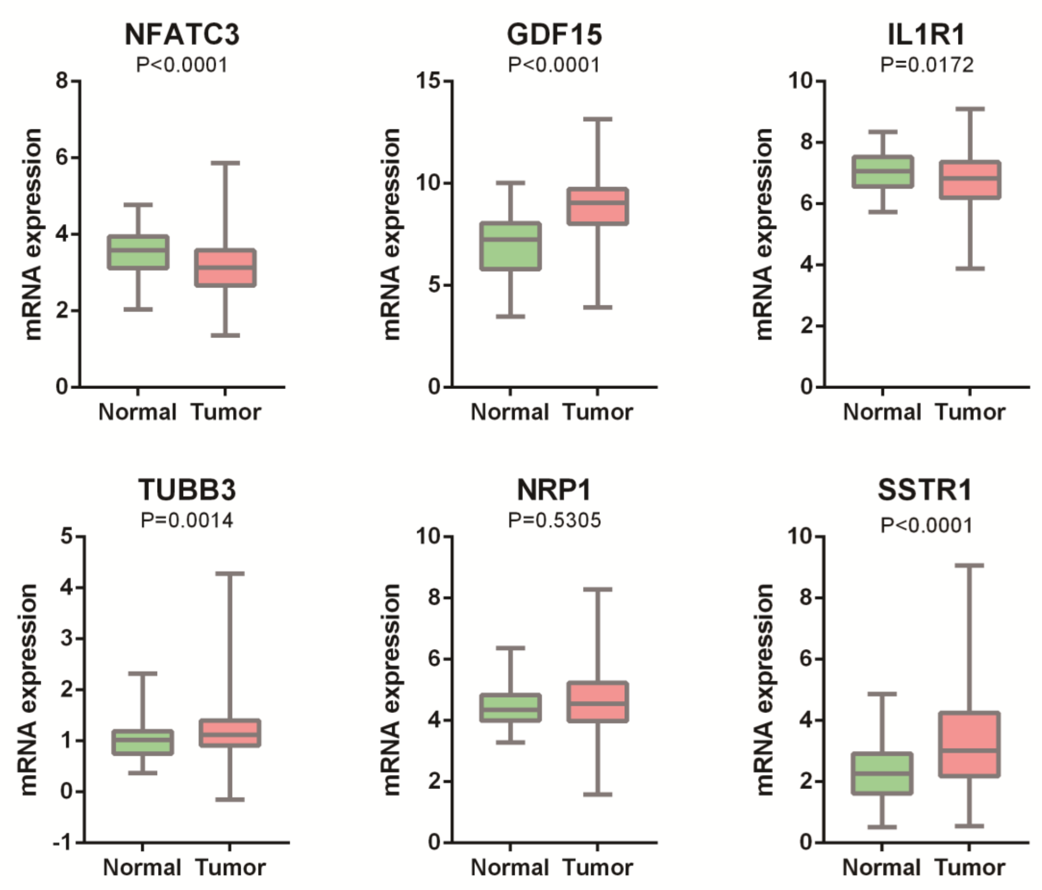

Table S1: Clinicopathological characteristics of prostate cancer patients in the training cohort and validation cohort.

Characteristics	Groups	Training cohort GSE70779	Validation cohort TCGA
		**N=92**	**N=379**
**Age**	≤65	NA	274
	>65	NA	105
	NM	92	0
**GS**	≤6	20	42
	7	55	191
	≥8	15	146
	NM	2	0
**PSA**	<10	56	NA
	≥10	34	NA
	NM	2	379
**Clinical stage (T)**	<T2b	75	180
	≥T2b	14	131
	NM	3	68
**Surgical margins**	Positive	42	NA
	Negative	50	NA
	NM	0	379

GS: Gleason score; PSA: prostate-specific antigen; NM: not mentioned; NA: not available.
